# Stereoassembled V_2_O_5_@FeOOH Hollow Architectures with Lithiation Volumetric Strain Self-Reconstruction for Lithium-Ion Storage

**DOI:** 10.34133/2020/2360796

**Published:** 2020-04-08

**Authors:** Yao Zhang, Kun Rui, Aoming Huang, Ying Ding, Kang Hu, Wenhui Shi, Xiehong Cao, Huijuan Lin, Jixin Zhu, Wei Huang

**Affiliations:** ^1^Key Laboratory of Flexible Electronics (KLOFE) & Institute of Advanced Materials (IAM), Nanjing Tech University (NanjingTech), 30 South Puzhu Road, Nanjing 211816, China; ^2^Center for Membrane and Water Science & Technology, Ocean College, Zhejiang University of Technology, Hangzhou 310014, China; ^3^College of Materials Science and Engineering, Zhejiang University of Technology, 18 Chaowang Road, Hangzhou, Zhejiang 310014, China; ^4^Shaanxi Institute of Flexible Electronics (SIFE), Northwestern Polytechnical University (NPU), 127 West Youyi Road, Xi'an 710072, China

## Abstract

Vanadium oxides have recently attracted widespread attention due to their unique advantages and have demonstrated promising chemical and physical properties for energy storage. This work develops a mild and efficient method to stereoassemble hollow V_2_O_5_@FeOOH heterostructured nanoflowers with thin nanosheets. These dual-phased architectures possess multiple lithiation voltage plateau and well-defined heterointerfaces facilitating efficient charge transfer, mass diffusion, and self-reconstruction with volumetric strain. As a proof of concept, the resulting V_2_O_5_@FeOOH hollow nanoflowers as an anode material for lithium-ion batteries (LIBs) realize high-specific capacities, long lifespans, and superior rate capabilities, e.g., maintaining a specific capacity as high as 985 mAh g^−1^ at 200 mA g^−1^ with good cyclability.

## 1. Introduction

Featuring high theoretical lithium storage capacity, considerable structural versatility and appealing electrochemical reactivity, transition metal oxides (TMOs, e.g., M_*x*_O_*y*_, M=Cu, Fe, Co, Ni, Mn, etc.) have received tremendous interest for potential energy storage applications [1–5]. Moreover, TMOs are endowed with enhanced safety as anode materials for lithium-ion batteries (LIBs), as the lithium dendrite growth can be inhibited at a relatively higher lithiation reaction potential. Therefore, TMOs have been widely explored and designed with expected structures, compositions, and size [6–9]. Particularly, vanadium oxides can intercalate more lithium ions owing to the distinct feature of multiple oxidation states (V^2+^ to V^5+^) [10–14]. In practice, despite the high theoretic capacity as both anode and cathode materials for LIBs, low electrical conductivity and large volumetric strains of V_2_O_5_ inevitably render poor rate capability and cycling stability [15–17]. To address these issues, carbon modification has been one of the most intuitive approaches to promote the electron transport for numerous TMOs-based electrode materials [18–20].

Alternatively, developing well-defined structures has also demonstrated great promise for boosting the electrochemical properties including enhanced capacity as well as desired stability [21–24]. For example, hierarchical assemblies consisting of nanosized building blocks, i.e., one-dimensional (1D) or two-dimensional (2D) subunits, are provided with combined merits of further enlarged contact area with electrolyte and reduced charge/mass diffusion pathways [25–28]. However, sacrificial templates associated with multistep procedures are usually involved for carefully designed complex structures. Furthermore, heterostructures through coupling versatile species are believed to offer more opportunities for various areas, which results from regulated electronic structures and intriguing synergistic effect at well-defined heterointerfaces [29–33]. More importantly, rational integration of functional components with consideration of their intrinsic redox properties allows tailored electrochemical behaviors for energy-based applications [34, 35]. To date, efficient construction of heterostructured electrodes based on V_2_O_5_ with tunable complex nanostructures remains a big challenge.

Herein, we demonstrate an efficient and facile method to stereoassemble dual-phased architectures of V_2_O_5_@FeOOH under a mild condition. Hierarchical V_2_O_5_@FeOOH heterostructures with well-defined morphology and composition are composed of highly connected ultrathin nanosheets, featuring well-defined heterointerfaces and multiple lithiation voltage plateau. Specifically, the V_2_O_5_@FeOOH with hollow nanoflower structures are well presented, enabling efficient charge transfer, mass diffusion, and self-reconstruction with volumetric strain. As a proof-of-concept demonstration, the V_2_O_5_@FeOOH hollow nanoflower anode delivers boosted Li-storage properties including high-specific capacities, long lifespans, and superior rate capabilities. Remarkably, a high-specific capacity of 985 mAh g^−1^ at 200 mA g^−1^ is achieved with good cyclability.

## 2. Results and Discussion

**Figure 1:**
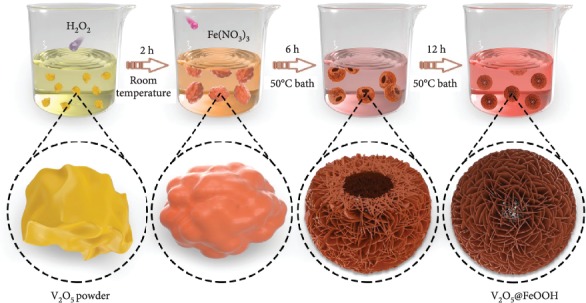
Schematic illustration for the stereoassembled V_2_O_5_@FeOOH hollow heterostructure.

The facile synthetic procedure of dual-phased hollow architectures is illustrated in Figure 1. The stereoassembled V_2_O_5_@FeOOH hollow heterostructures are obtained by using commercial V_2_O_5_ as the starting material under a mild condition (see the Experimental section for details). Specifically, irregular bulk V_2_O_5_ powders (Figure S1) are first dispersed in deionized water, followed by dropwise addition of hydrogen peroxide (H_2_O_2_) at room temperature to form a uniform solution. During which, the dissolution of V_2_O_5_ with the presence of H_2_O_2_ results in the formation of complex vanadium-based intermediates [36]. Afterward, the self-assembly of V_2_O_5_ nanosheets into well-defined hierarchical hollow architecture can be realized with the assistance of iron(III) nitrate nonahydrate (Fe(NO_3_)_3_·9H_2_O) at 50°C by controlling the reaction time (Figures S2-S6). Importantly, Fe(NO_3_)_3_ in this case acts as the shape-directing agent to manipulate the morphology, as bare 2D nanosheets with large lateral size and smooth surface can be obtained without the addition of Fe(NO_3_)_3_ (Figure S7). Furthermore, Fe(NO_3_)_3_ contributes to the nanoscale heterostructuring with optimized composition through in situ deposition of FeOOH as well. Lastly, a reddish-brown V_2_O_5_@FeOOH product can be harvested after a vacuum freeze-drying process.

**Figure 2:**
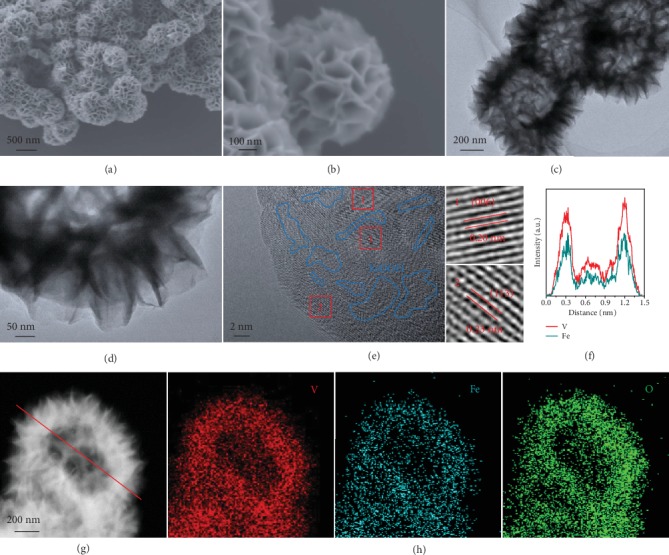
Microstructure characterization and composition analysis of hollow V_2_O_5_@FeOOH heterostructure. (a, b) FESEM images of V_2_O_5_@FeOOH. (c, d) TEM images of V_2_O_5_@FeOOH. (e) HRTEM image of V_2_O_5_@FeOOH. (f) Dark-field STEM-EDS line scan profile of V_2_O_5_@FeOOH. (g, h) Dark-field TEM image and corresponding elemental mapping images of V_2_O_5_@FeOOH with red for V, cyan for Fe, and green for O.

The morphology and structure of the V_2_O_5_@FeOOH heterostructures are investigated by field emission scanning electron microscopy (FESEM). As presented by the panoramic image in Figure 2(a), these V_2_O_5_@FeOOH particles exhibit a flower-like sphere structure with high uniformity. A closer FESEM examination revealed the hierarchical architecture of V_2_O_5_@FeOOH, which are assembled from nanosheet subunits with a small thickness of about 10 nm (Figure 2(b)). Interestingly, a well-defined hollow interior of approximately 300 nm can be clearly observed from the transmission electron microscopy (TEM) image (Figure 2(c)). At a higher magnification, the thin character of these wrinkled nanosheets is suggested by their high transparency under the electron beam (Figure 2(d)). Notably, a set of diffraction spots well fit the orthorhombic V_2_O_5_ lattice structure, which is viewed along the [010] zone axis according to the selected area electron diffraction (SAED) data (Figure S8). In the high-resolution TEM (HRTEM) image (Figure 2(e)), the crystalline nanosheet shows typical lattice spacings of 0.23 nm and 0.20 nm, corresponding to the (113) and (006) planes of V_2_O_5_·1.6H_2_O. Meanwhile, notably discontinuous lattice fringes can be observed, which can be readily attributed to the vertical coverage of amorphous FeOOH domains. Moreover, scanning transmission electron microscopy-energy dispersive spectroscopy (STEM-EDS) elemental mapping images of V_2_O_5_@FeOOH corresponding to the dark-field image (Figure 2(g)) reveal the homogeneous distribution of V, Fe, and O (Figure 2(h)). As demonstrated by the EDS line scan profile in Figure 2(f), both V and Fe signals exhibit relatively higher intensity at the edge region as compared to the core region, further validating the formation of hollow structure.

**Figure 3:**
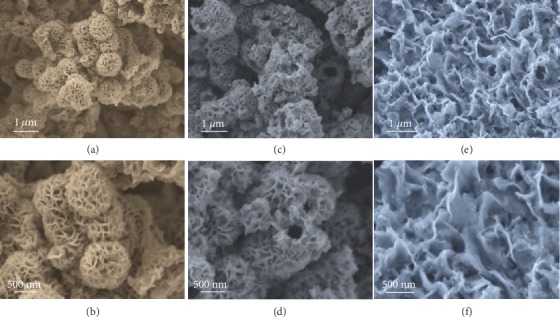
FESEM images of V_2_O_5_@FeOOH heterostructures obtained by altering the mass ratio of Fe(III) precursor and V_2_O_5_. (a, b) V_2_O_5_@FeOOH-1, (c, d) V_2_O_5_@FeOOH-2, and (e, f) V_2_O_5_@FeOOH-5.

It is worth noting that the configuration of V_2_O_5_@FeOOH heterostructure can be precisely tuned by varying the amount of Fe(III) precursor (Figure 3). Interestingly, the flower-like V_2_O_5_@FeOOH spheres expanded significantly with the increasing amount of Fe(III) precursor. In sharp contrast to V_2_O_5_@FeOOH-1 that is obtained by using Fe(NO_3_)_3_ and V_2_O_5_ with a mass ratio of 1 : 1 (Figures 3(a) and 3(b)), V_2_O_5_@FeOOH-2 evolves into irregular assembly of nanosheets, exhibiting open cavities as surrounding 2D building blocks tend to spread out (Figures 3(c) and 3(d)). Importantly, the hierarchical hollow structure hardly maintains when further increasing the mass ratio of Fe(NO_3_)_3_ to V_2_O_5_ (V_2_O_5_@FeOOH-5). Highly interconnected 3D architectures can be obtained, which are constructed from randomly assembled nanosheets with rough surfaces (Figures 3(e) and 3(f)). For comparison, bare FeOOH particles can be obtained by the same procedure except for the presence of V_2_O_5_ (Figure S9).

**Figure 4:**
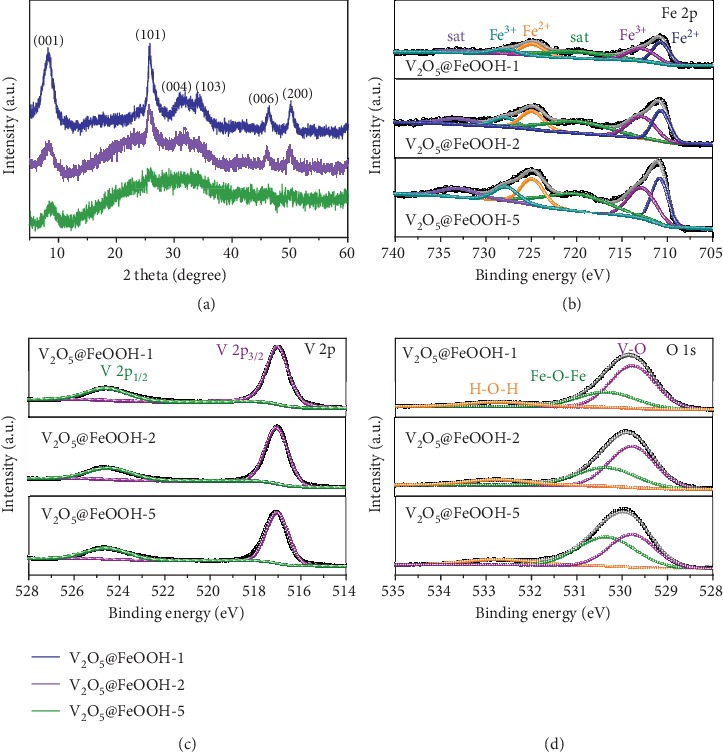
(a) XRD patterns of V_2_O_5_@FeOOH heterostructures. (b–d) XPS analysis of V_2_O_5_@FeOOH heterostructures: (b) Fe 2p, (c) V 2p, and (d) O 1s spectra.

The crystal structure of as-synthesized samples is then determined through X-ray diffraction (XRD) measurement (Figure 4(a)). For V_2_O_5_@FeOOH-1, the diffraction peaks at 8.3°, 25.9°, 31.1°, 34.2°, 46.4°, and 50.3° can be well assigned to (001), (101), (004), (103), (006), and (200) reflections of orthorhombic structured V_2_O_5_·1.6H_2_O (JCPDS no. 40-1296). On the other hand, no characteristic peaks belonging to FeOOH are detected, which should be attributed to its amorphous feature [37]. The lower crystallinity of V_2_O_5_@FeOOH-2 and V_2_O_5_@FeOOH-5 as indicated by the disappearing of well-defined diffraction peaks further suggest the increased content of amorphous phase within the V_2_O_5_@FeOOH hybrids. The chemical composition and bonding state of V_2_O_5_@FeOOH are also investigated by X-ray photoelectron spectroscopy (XPS). The high-resolution Fe 2p spectra (Figure 4(b) and Figure S10) confirm the presence of Fe species as FeOOH [38, 39], with the coexistence of Fe^2+^ and Fe^3+^ at 710.7/724.8 eV and 712.8/727.8 eV, respectively. The peak intensity for Fe 2p_1/2_, 2p_3/2_, and satellite together enlarged from V_2_O_5_@FeOOH-1 to V_2_O_5_@FeOOH-5, in accordance with the increasing incorporation of Fe species within the heterostructure (Table S1). The V 2p region spectra of V_2_O_5_@FeOOH are analyzed, exhibiting characteristic peaks centered at 524.6 and 517.0 eV for V^5+^ components of V_2_O_5_ (Figure 4(c)) [40–42]. Besides, a negative shift of binding energy (~0.8 eV) for both V 2p_1/2_ and 2p_3/2_ bands are achieved as compared to bare V_2_O_5_ (Figure S11), indicating the strong electronic interaction at the V_2_O_5_/FeOOH interfaces. Meanwhile, the deconvoluted O 1s core level spectra show two dominant peaks that can be assigned to V-O and Fe-O-Fe bonds [43], which reveal varied portions of the Fe-O-Fe at 530.4 eV accordingly (Figure 4(d)).

**Figure 5:**
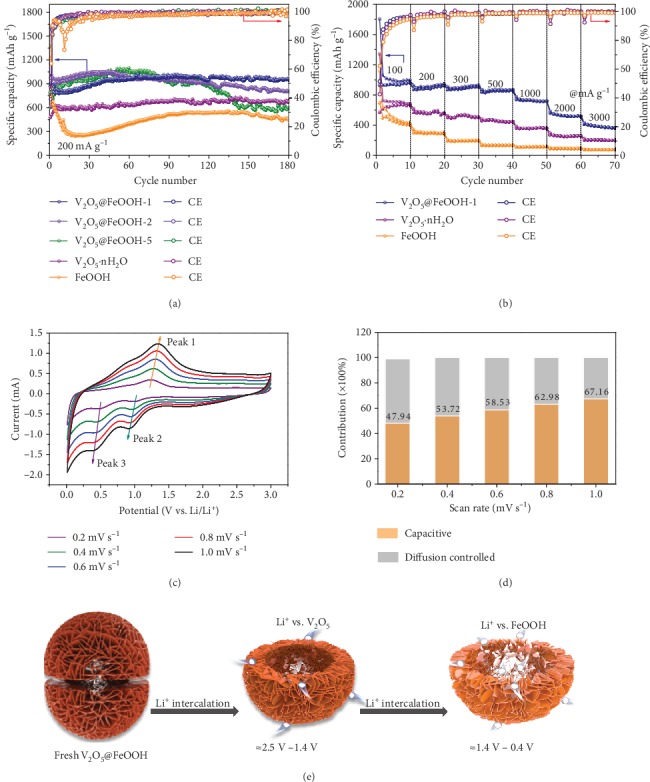
Electrochemical properties in Li^+^ storage. (a) Cycling performance of V_2_O_5_@FeOOH-1, V_2_O_5_@FeOOH-2, V_2_O_5_@FeOOH-5, V_2_O_5_·nH_2_O, and FeOOH at 200 mA g^−1^. (b) The rate-capacity performance of V_2_O_5_@FeOOH-1, V_2_O_5_·nH_2_O, and FeOOH from 100 to 3000 mA g^−1^. (c) Cyclic voltammetry curves of V_2_O_5_@FeOOH-1 at a series of scan rates from 0.2 to 1.0 mV s^−1^. (d) Normalized contribution ratio of capacitive (orange) and diffusion-controlled (gray) capacities at various scan rates. (e) Schematic illustration for V_2_O_5_@FeOOH-1 electrode during Li^+^ intercalation process.

As a proof of concept, the electrochemical properties of heterostructured V_2_O_5_@FeOOH as LIB anodes are examined (Figure 5). The initial Coulombic efficiency (CE) is around 50% for V_2_O_5_@FeOOH (e.g., 50% for V_2_O_5_@FeOOH-1, 53% for V_2_O_5_@FeOOH-2, and 43% for V_2_O_5_@FeOOH-5), which can be attributed to the irreversible capacity loss due to the formation of solid electrolyte interphase (SEI) film on the electrode surface [44]. After a typical activation process for 50 cycles [45], remarkable reversible capacities as high as 985, 1016, and 1030 mAh g^−1^ at a current density of 200 mA g^−1^ are delivered by V_2_O_5_@FeOOH-1, V_2_O_5_@FeOOH-2, and V_2_O_5_@FeOOH-5, respectively, reaching the CE of nearly 100%. The desirable Li-storage capacities surpassing bare V_2_O_5_ (635 mAh g^−1^) and FeOOH (342 mAh g^−1^) manifest the superiority of dual-phased heterostructures constructed by 2D building blocks. Different from V_2_O_5_@FeOOH-2 and V_2_O_5_@FeOOH-5 with capacity fading after ca. 100 cycles, 96% capacity is retained for V_2_O_5_@FeOOH-1 after 180 cycles, which can be further attributed to its well-defined hollow configuration. The 3D hierarchical structure integrity of V_2_O_5_@FeOOH-1 can be maintained during repeated Li^+^ insertion and exaction as confirmed by postmortem FESEM images in Figure S12. Of note, the cycling stability of V_2_O_5_@FeOOH-1 can be further highlighted at a higher current density of 2000 mA g^−1^, i.e., 494 mAh g^−1^ after 300 cycles with a high capacity retention of 95% (Figure S13).

Importantly, rate performance of the V_2_O_5_@FeOOH-1 anode was investigated at different current densities from 100 mA g^−1^ to 3000 mA g^−1^ (Figure 5(b)). Reversible capacities of 992, 947, 906, 856, 714, 519, and 366 mAh g^−1^ are achieved at current densities of 100, 200, 300, 500, 1000, 2000, and 3000 mA g^−1^, respectively. Cyclic voltammetry (CV) profiles at different scan rates from 0.2 to 1.0 mV s^−1^ were therefore recorded to verify the electrochemical kinetics of V_2_O_5_@FeOOH-1 for Li-storage (Figure 5(c)). Negligible peak shifts are observed as the scan rates increase, suggesting small polarization as well as desirable kinetics of V_2_O_5_@FeOOH-1 at high rates. The relationship of log (*i*, peak current) versus log (*v*, scan rate) was plotted to give the fitted values of slope *b* (Figure S14), which provides insights into the charge storage mechanism according to the power-law formula *i* = *av*^*b*^ [46]. Specifically, a *b* value of 0.5 represents an ideal diffusion-controlled process, whereas 1.0 indicates a surface capacitive-controlled one. The calculated *b* values are 0.74 for anodic peak 1 and 0.83 and 0.72 for the cathodic peaks 2 and 3, respectively, demonstrating a partial capacitive-controlled behavior of V_2_O_5_@FeOOH-1. Quantitative analysis further reveals that the ratios of capacitive contribution gradually improve upon increasing the scan rates (Figure 5(d), see calculation details and Figure S15 in Supplementary Materials), which reaches 67.16% at 1.0 mV s^−1^ (Figure S16). The considerable pseudocapacitive contributions can be readily attributed to the presence of abundant amorphous domains and numerous grain boundaries on the surface of highly exposed nanosheets. These electrochemically active sites are undoubtedly favorable for boosting Li-storage capacity and especially rate performance of V_2_O_5_@FeOOH-1 hybrid. Furthermore, Nyquist plots demonstrated reduced charge transfer resistances (*R*_ct_) of V_2_O_5_@FeOOH-1 (114.7 *Ω*) as compared to FeOOH (125.5 *Ω*), and V_2_O_5_·nH_2_O (181.0 *Ω*), which was fitted according to the equivalent circuit (Figure S17 and Table S2). Moreover, the plot slope of *Z*′ vs. *ω*^−1/2^ can be obtained to illustrate the speed of lithium diffusion, which is calculated to be 83.7 for V_2_O_5_@FeOOH-1, 171.3 for FeOOH, and 128.2 for V_2_O_5_·nH_2_O (Figure S18). Therefore, the lithium diffusion coefficients at 25°C are calculated to be 5.98 × 10^−14^, 5.28 × 10^−15^, and 2.55 × 10^−14^ cm^2^ s^−1^ for V_2_O_5_@FeOOH-1, FeOOH, and V_2_O_5_·nH_2_O, respectively. The results indicate that the V_2_O_5_@FeOOH-1 hybrid can provide more accessible pathway for charge transfer due to the sufficient heterointerface and accelerated lithium diffusion, resulting in enhanced Li-storage performance.

Additionally, Li-storage performance of V_2_O_5_@FeOOH-1 are superior to that of many other reported vanadium-based oxide materials (Table S3). The substantially optimized electrochemical properties can be ascribed to the rational construction of V_2_O_5_@FeOOH heterostructures with well-defined 3D configurations featuring intriguing interfaces and synergistic effects. Specifically, the V_2_O_5_ nanosheets in the first place serve as ideal building blocks with promoted Li^+^/electron transport owing to their ultrathin feature. After introducing amorphous FeOOH, the Li intercalation process can be readily tailored due to the distinct lithiation voltage plateau of FeOOH and V_2_O_5_ as schematically illustrated in Figure 5(e). Upon discharge, the lithiation of V_2_O_5_ occurs at a higher voltage (2.5-1.4 V) [47], during which the volumetric expansion can be effectively restrained owing to the presence of amorphous FeOOH as surface buffers. Afterwards, FeOOH further contributes considerable capacity at lower voltages (1.4-0.4 V) [48], which is also confined within the inner space provided by interconnected nanosheets. Apart from the strain self-reconstruction enabled by the synergy between V_2_O_5_ and FeOOH, the hollow configuration is highly appealing for strain accommodation and cycling stability during repeated lithiation and delithiation. More importantly, extra Li^+^ storage sites together with diffusion channels are created where the presence of amorphous FeOOH domains induce abundant grain boundaries of V_2_O_5_ and substantial interfacial area, contributing to boosted reaction activity and enhanced Li-storage capacity.

## 3. Conclusions

In summary, a novel hollow V_2_O_5_@FeOOH heterostructured nanoflower has been stereoassembled via an efficient and mild method. These nanoflowers composed of well-connected ultrathin nanosheets demonstrate superior advantages including enlarged electrode-electrolyte contact, facilitated charge transfer, and accelerated mass diffusion for LIBs. Owing to the multiple lithiation voltage plateau and well-defined heterointerfaces, the hollow V_2_O_5_@FeOOH nanoflowers deliver excellent lithium storage properties with a high reversible capacity (985 mAh g^−1^ at 200 mA g^−1^ after 180 cycles) and remarkable cycling stability (95% capacity retention at 2000 mA g^−1^ after 300 cycles). It is expected that the present result can be further extended to optimize other TMO-based materials and shed lights on the development of future energy applications.

## 4. Material and Methods

### 4.1. Preparation of V_2_O_5_@FeOOH Hollow Heterostructures

0.364 g commercial V_2_O_5_ powder was mixed with 25 mL DI water and 5 mL H_2_O_2_ (30 wt%) under stirring for 2 h to form a dark-red solution at room temperature. Fe(NO_3_)_3_·9H_2_O was then added to the above mixture under stirring, accompanied with water bath at 50°C overnight. The reddish-brown precipitates were collected by centrifugation and washed with water and ethanol several times. Finally, V_2_O_5_@FeOOH was obtained by a freeze-drying process. By varying the amount of Fe(NO_3_)_3_·9H_2_O, a series of V_2_O_5_@FeOOH heterostructures were prepared and denoted as V_2_O_5_@FeOOH-1, V_2_O_5_@FeOOH-2, and V_2_O_5_@FeOOH-5. Take V_2_O_5_@FeOOH-2 as an example, the mass ratio of Fe(NO_3_)_3_·9H_2_O to V_2_O_5_ is 2 : 1.

### 4.2. Preparation of V_2_O_5_·nH_2_O

0.364 g commercial V_2_O_5_ was dispersed in 25 mL DI water under stirring, followed by the addition of 5 mL H_2_O_2_ (30%) to form a dark-red solution. After aging at room temperature for 2 h, the mixture was maintained at 50°C under stirring overnight to form a hydrogel. Finally, V_2_O_5_·nH_2_O was collected after a freeze-drying process.

### 4.3. Preparation of FeOOH

0.364 g Fe(NO_3_)_3_·9H_2_O was first dissolved in 25 mL DI water containing 5 mL H_2_O_2_ (30%) under stirring at room temperature. The solution was then maintained at 50°C overnight. The precipitates were rinsed with water and ethanol several times by centrifugation. Finally, the as-obtained product was collected after freeze-drying overnight.

### 4.4. Materials Characterization

FESEM (JEOL, JSM-7600F) and TEM (JEOL, JEM-2100F) coupled with EDS spectroscopy were used to investigate the morphology, structure and composition of the as-obtained samples. Crystal phases of the obtained samples were identified using XRD (Rigaku, SmartLab with Cu K*α* radiation). XPS (Thermo-VG Scientific, ESCALAB 250) was employed to characterize the compositions and valence states of the products.

### 4.5. Electrochemical Measurements

The electrochemical measurements of the as-prepared active materials were performed according to previously reported procedures [49]. Specifically, CR2032-type coin cells were assembled in an argon-filled glove box with the contents of moisture and oxygen less than 0.5 ppm. 70 wt% of the product (e.g., V_2_O_5_@FeOOH-1, FeOOH, and V_2_O_5_·nH_2_O) was mixed with 20 wt% multiwalled carbon nanotube and 10 wt% polyvinylidene difluoride into NMP to prepare the working electrode. The as-obtained slurry was uniformly pasted on the Cu foil with a mass loading of about 1 mg cm^−2^ and dried under vacuum at 60°C for 24 h to remove the solvent. For the LIBs test, the lithium metal foil was used as the counter/reference electrode, 1.0 M LiPF_6_ dissolved into a mixture of ethylene carbonate (EC), dimethyl carbonate (DMC), and ethyl methyl carbonate (EMC) (EC/DMC/EMC, 1 : 1 : 1, *v*/*v*/*v*) was used as electrolyte, and Celgard 2400 membrane was used as the separator. The galvanostatic charge-discharge tests at various current densities were conducted with a battery testing system (NEWARE, CT-4008) under a voltage range of 0.01 to 3.0 V. The CV curves were obtained on a Bio-logic (VMP-300) electrochemical workstation.

## Supplementary Materials

Supplementary Materials Figure S1: SEM images of commercial V_2_O_5_ powder. Figure S2: SEM images of the vanadium-based intermediate formed immediately after introducing Fe(NO_3_)_3_ at 50°C. Figure S3: SEM images of the vanadium-based intermediate obtained after reacting with Fe(NO_3_)_3_ at 50°C for 3 h. Figure S4: SEM images of the vanadium-based intermediate obtained after reacting with Fe(NO_3_)_3_ at 50°C for 6 h. Figure S5: SEM images of the vanadium-based intermediate obtained after reacting with Fe(NO_3_)_3_ at 50°C for 12 h. Figure S6: The XRD patterns of the vanadium-based intermediate. Figure S7: SEM, TEM images, and corresponding elemental mapping images of pure V_2_O_5_·nH_2_O. Figure S8: the SAED pattern for V_2_O_5_@FeOOH-1. Figure S9: SEM, TEM images, and corresponding elemental mapping images of pure FeOOH. Figure S10: Fe 2p XPS spectrum of FeOOH. Figure S11: XPS analysis of pure V_2_O_5_·nH_2_O. Figure S12: SEM images of V_2_O_5_@FeOOH-1 after 180 cycles at a current density of 200 mA g^−1^. Figure S13: cycling performance of V_2_O_5_@FeOOH-1, V_2_O_5_·nH_2_O, and FeOOH at a current density of 2000 mA g^−1^. Figure S14: *b* value determined by the relationship between scan rate and peak current. Figure S15: plots of *v*^1/2^ vs. *i*/*v*^1/2^ for calculating constants k_1_ and k_2_ at different potentials. Figure S16: capacitive contribution to charge storage of V_2_O_5_@FeOOH-1 at 1.0 mV s^−1^. Figure S17: Nyquist plots of V_2_O_5_@FeOOH-1, FeOOH, and V_2_O_5_·nH_2_O. Figure S18: the relationships between *w*^−1/2^ and *Z*′ in the low-frequency region of V_2_O_5_@FeOOH-1, FeOOH, and V_2_O_5_·nH_2_O. Table S1: the molar ratio of V and Fe for V_2_O_5_@FeOOH-1, V_2_O_5_@FeOOH-1, and V_2_O_5_@FeOOH-5 calculated based on XPS results. Table S2: fitted equivalent resistances for EIS results of V_2_O_5_@FeOOH-1, FeOOH, and V_2_O_5_·nH_2_O. Table S3: comparison of lithium storage properties of various vanadium-based oxide materials.
