# Supplementary material for: Stereoassembled V2O5@FeOOH Hollow Architectures with Lithiation Volumetric Strain Self-Reconstruction for Lithium-Ion Storage
Source: Research (Wash D C). 2020 Apr 8;2020:2360796. doi: 10.34133/2020/2360796 (PMC7168343; doi:10.34133/2020/2360796)
Supplement: Supplementary Materials — Figure S1: SEM images of commercial V2O5 powder. Figure S2: SEM images of the vanadium-based intermediate formed immediately after introducing Fe(NO3)3 at 50°C. Figure S3: SEM images of the vanadium-based intermediate obtained after reacting with Fe(NO3)3 at 50°C for 3 h. Figure S4: SEM images of the vanadium-based intermediate obtained after reacting with Fe(NO3)3 at 50°C for 6 h. Figure S5: SEM images of the vanadium-based intermediate obtained after reacting with Fe(NO3)3 at 50°C for 12 h. Figure S6: The XRD patterns of the vanadium-based intermediate. Figure S7: SEM, TEM images, and corresponding elemental mapping images of pure V2O5·nH2O. Figure S8: the SAED pattern for V2O5@FeOOH-1. Figure S9: SEM, TEM images, and corresponding elemental mapping images of pure FeOOH. Figure S10: Fe 2p XPS spectrum of FeOOH. Figure S11: XPS analysis of pure V2O5·nH2O. Figure S12: SEM images of V2O5@FeOOH-1 after 180 cycles at a current density of 200 mA g−1. Figure S13: cycling performance of V2O5@FeOOH-1, V2O5·nH2O, and FeOOH at a current density of 2000 mA g−1. Figure S14: b value determined by the relationship between scan rate and peak current. Figure S15: plots of v1/2 vs. i/v1/2 for calculating constants k1 and k2 at different potentials. Figure S16: capacitive contribution to charge storage of V2O5@FeOOH-1 at 1.0 mV s−1. Figure S17: Nyquist plots of V2O5@FeOOH-1, FeOOH, and V2O5·nH2O. Figure S18: the relationships between w−1/2 and Z′ in the low-frequency region of V2O5@FeOOH-1, FeOOH, and V2O5·nH2O. Table S1: the molar ratio of V and Fe for V2O5@FeOOH-1, V2O5@FeOOH-1, and V2O5@FeOOH-5 calculated based on XPS results. Table S2: fitted equivalent resistances for EIS results of V2O5@FeOOH-1, FeOOH, and V2O5·nH2O. Table S3: comparison of lithium storage properties of various vanadium-based oxide materials. [file 2360796.f1.docx]

Supplementary Materials

**Stereoassembled V_2_O_5_@FeOOH Hollow Architectures with Lithiation Volumetric Strain Self-Reconstruction for Lithium Ion Storage**

Yao Zhang^1^, Kun Rui^1^*, Aoming Huang^1^, Ying Ding^1^, Kang Hu^1^, Wenhui Shi^2^, Xiehong Cao^3^, Huijuan Lin^1^, Jixin Zhu^1^*, and Wei Huang^1,4^

*^1^Key Laboratory of Flexible Electronics (KLOFE) & Institute of Advanced Materials (IAM) Nanjing Tech University (NanjingTech), 30 South Puzhu Road, Nanjing 211816, P. R. China*

*^2^Center for Membrane and Water Science & Technology, Ocean College, Zhejiang University of Technology, Hangzhou 310014, P. R. China*

*^3^College of Materials Science and Engineering, Zhejiang University of Technology, 18 Chaowang Road, Hangzhou, Zhejiang 310014, P. R. China*

*^4^Shaanxi Institute of Flexible Electronics (SIFE), Northwestern Polytechnical University (NPU), 127 West Youyi Road, Xi’an 710072, P.R. China*

*Correspondence should be addressed to Kun Rui; [iamkrui@njtech.edu.cn](mailto:iamkrui@njtech.edu.cn) and Jixin Zhu; [iamjxzhu@njtech.edu.cn](mailto:iamjxzhu@njtech.edu.cn)


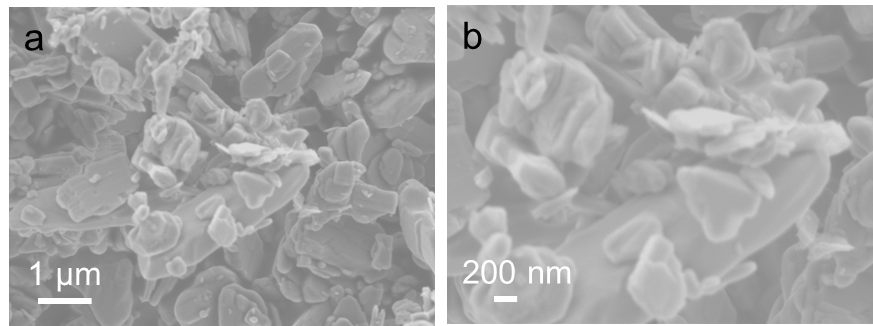


**Figure S1.** SEM images of commercial V_2_O_5_ powder.


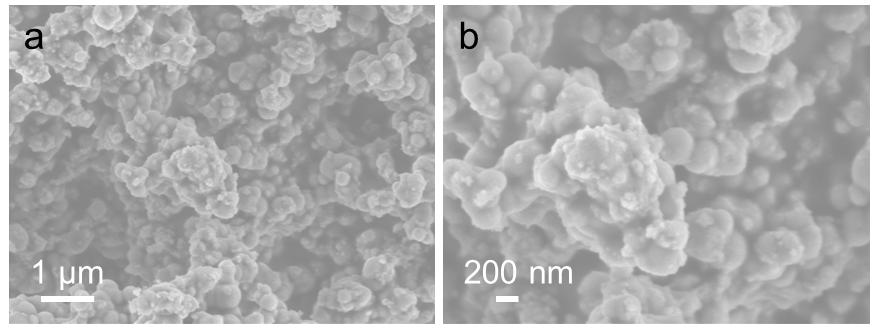


**Figure S2.** SEM images of the vanadium-based intermediate formed immediately after introducing Fe(NO_3_)_3_ at 50 °C.


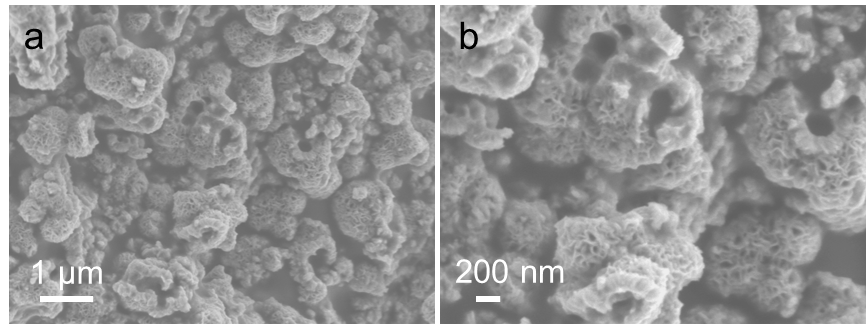


**Figure S3.** SEM images of the vanadium-based intermediate obtained after reacting with Fe(NO_3_)_3_ at 50 °C for 3h.


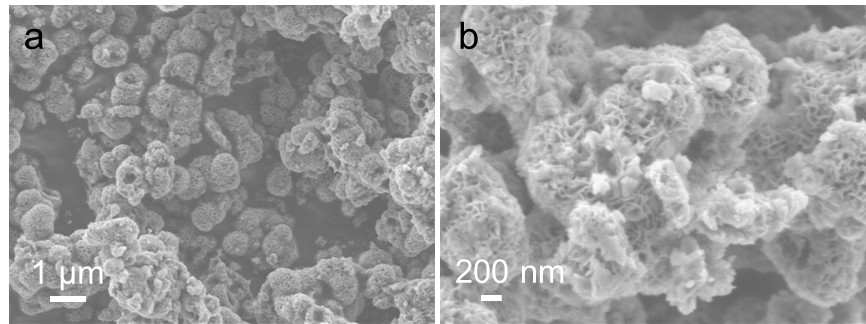


**Figure S4.** SEM images of the vanadium-based intermediate obtained after reacting with Fe(NO_3_)_3_ at 50 °C for 6h.


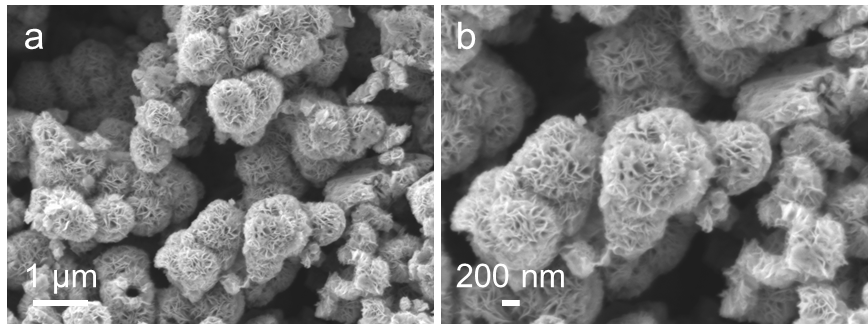


**Figure S5.** SEM images of the vanadium-based intermediate obtained after reacting with Fe(NO_3_)_3_ at 50 °C for 12h.


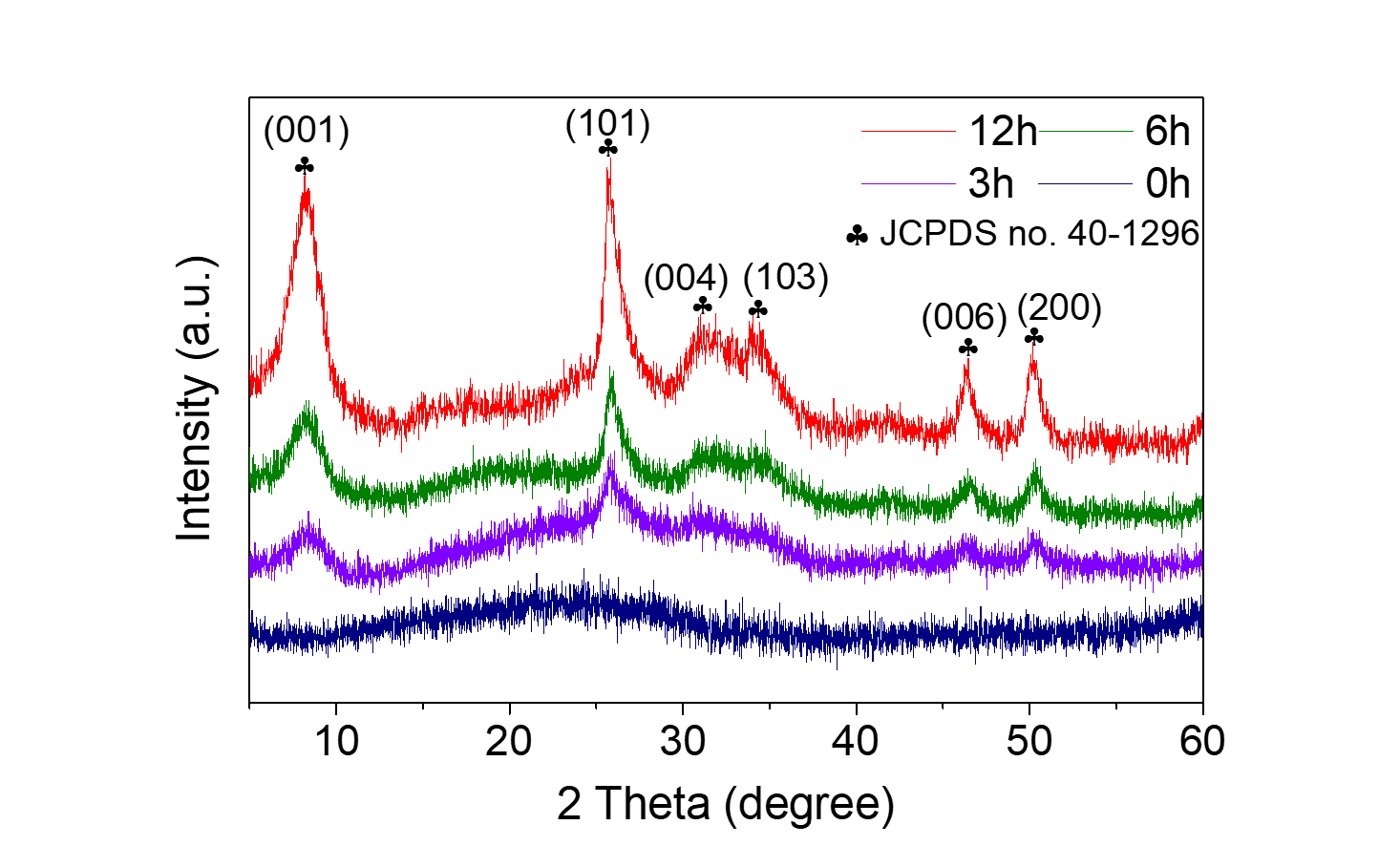


**Figure S6.** The XRD patterns of the vanadium-based intermediate obtained after reacting with Fe(NO_3_)_3_ at 50 °C during morphology evolution.


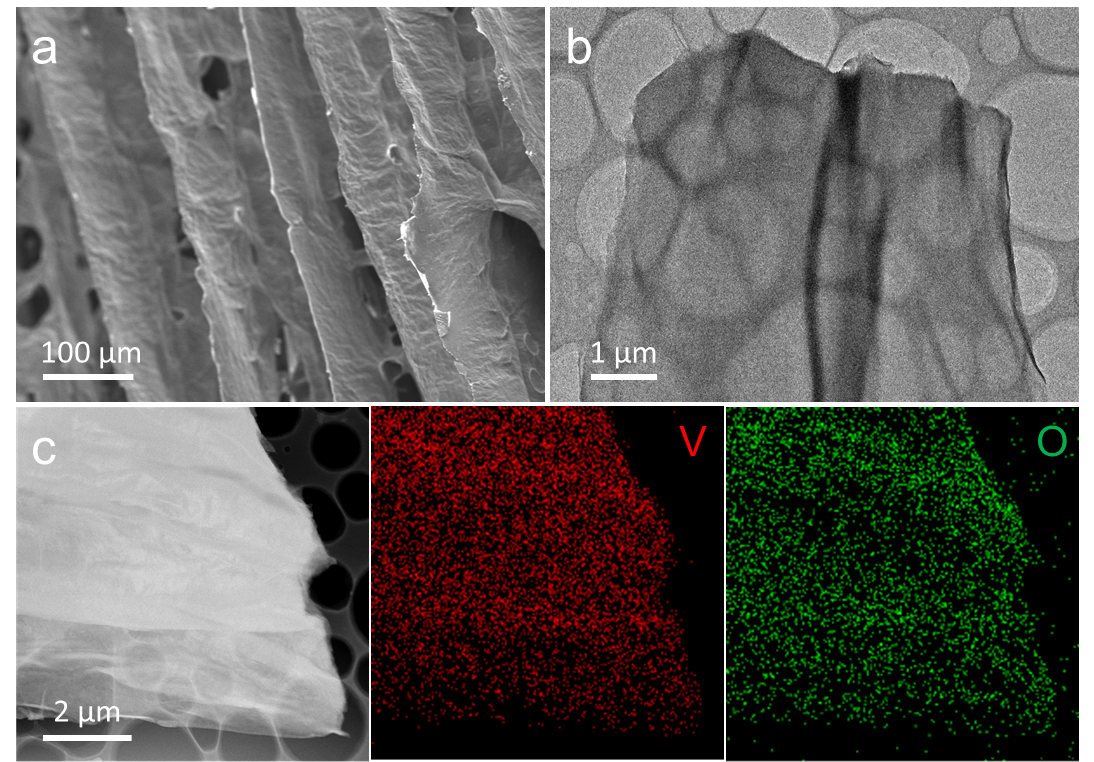


**Figure S7.** (a, b) SEM and TEM images of pure V_2_O_5_·nH_2_O. (c) Dark-field TEM image and corresponding elemental mapping images of V_2_O_5_·nH_2_O with red for V, and green for O.

**
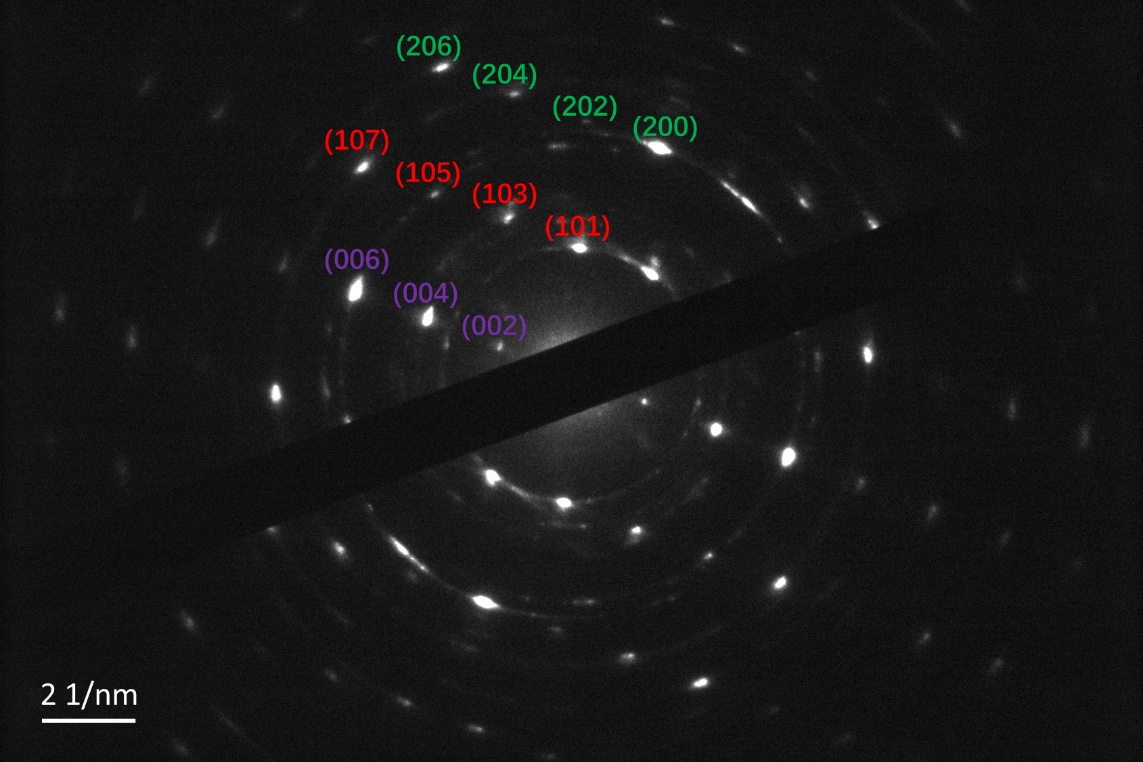
**

**Figure S8.** The SAED pattern for V_2_O_5_@FeOOH-1.


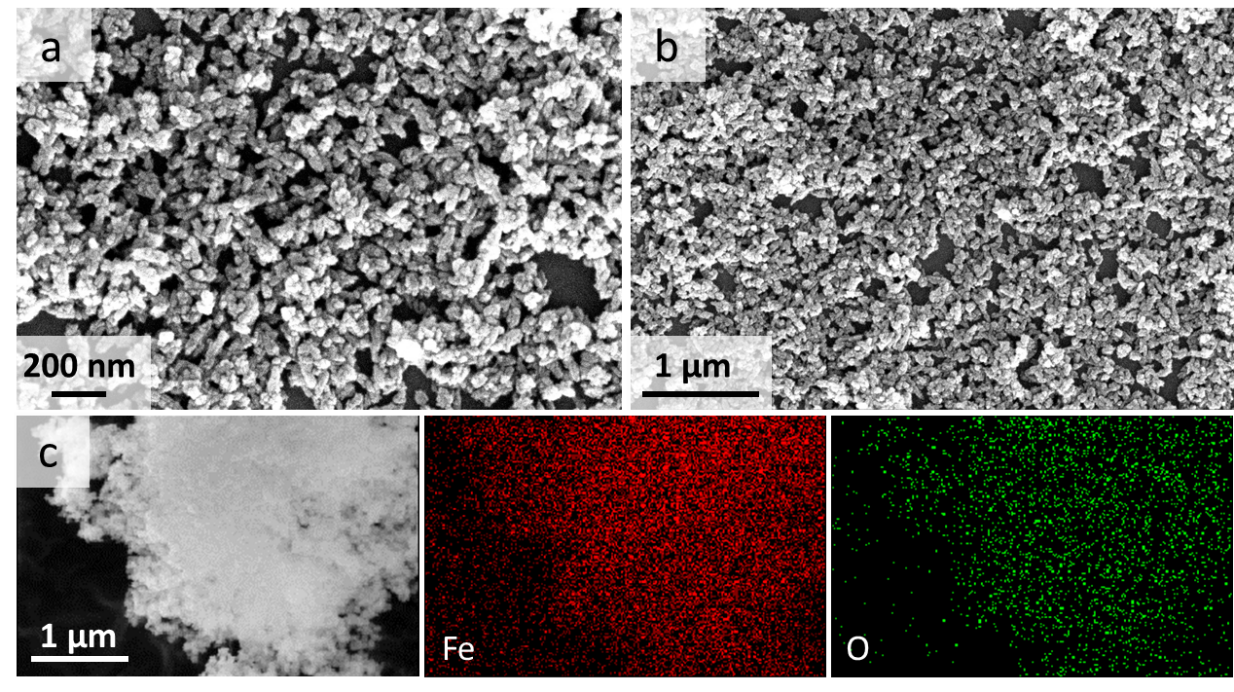


**Figure S9.** (a, b) SEM images of pure FeOOH. (c) Dark-field TEM image and corresponding elemental mapping images of FeOOH with red for Fe, and green for O.


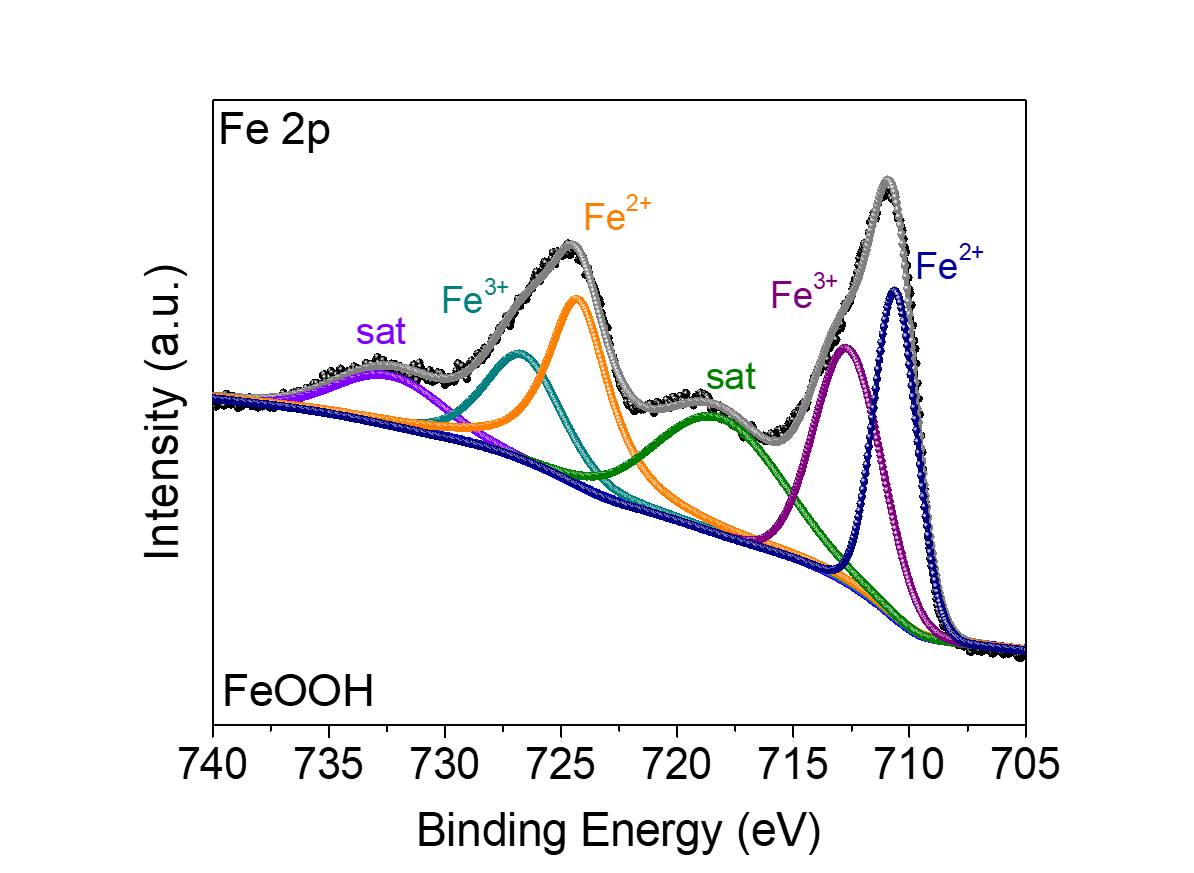


**Figure S10.** Fe 2p XPS spectrum of FeOOH.


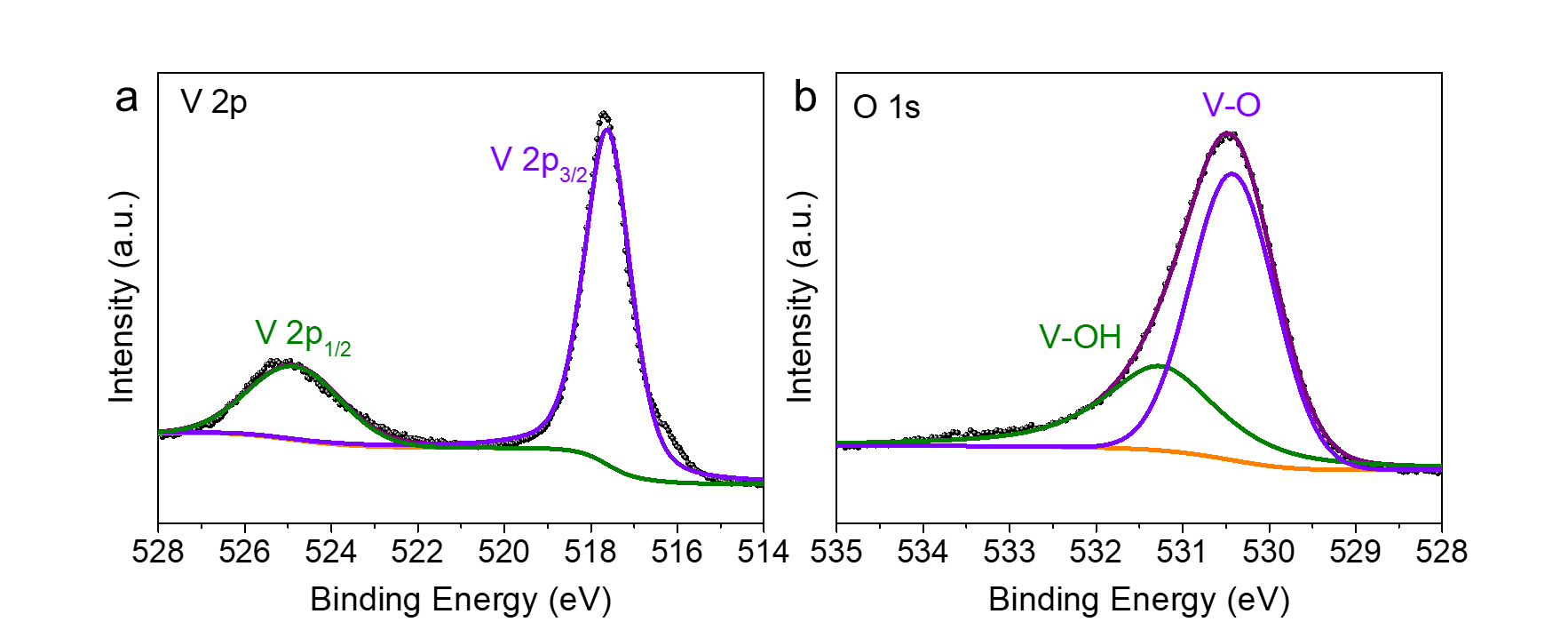


**Figure S11.** XPS analysis of pure V_2_O_5_·nH_2_O: (a) V 2p and (b) O 1s spectra.

**
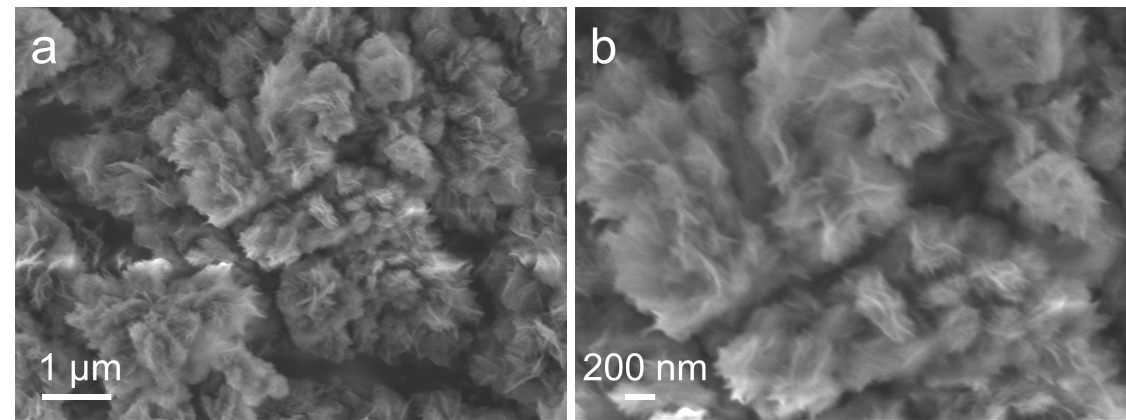
**

**Figure S12.** SEM images of V_2_O_5_@FeOOH-1 after 180 cycles at a current density of 200 mA g^−1^.


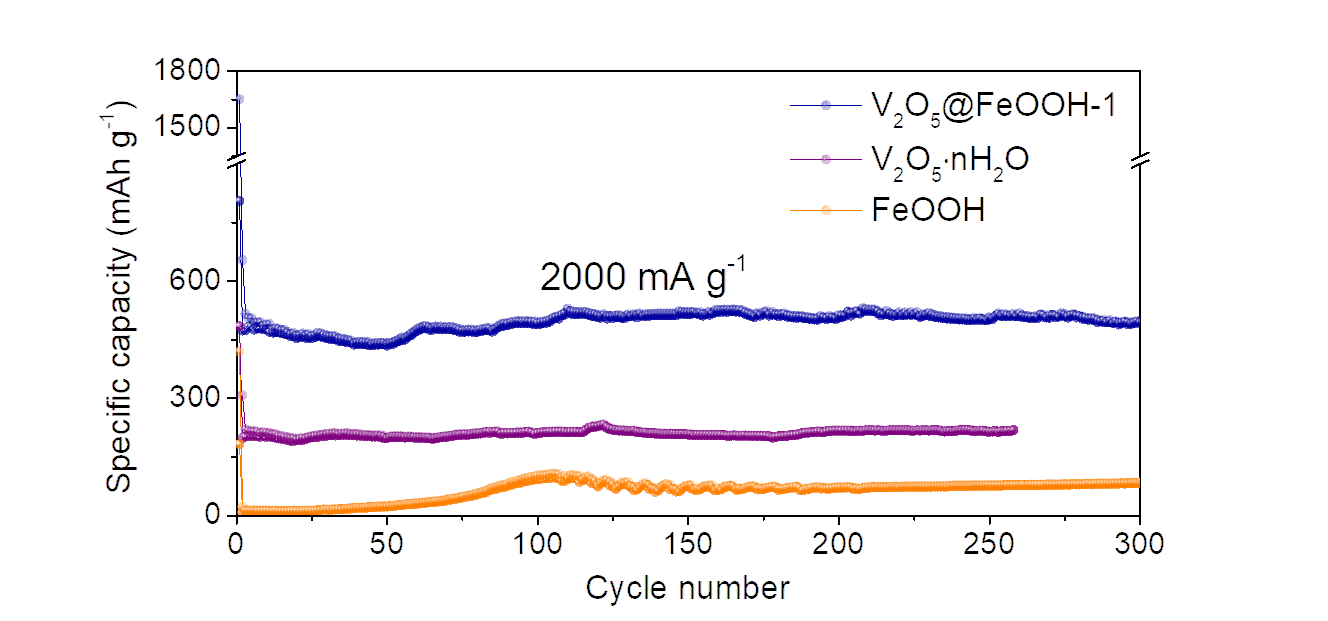


**Figure S13.** Cycling performance of V_2_O_5_@FeOOH-1, V_2_O_5_·nH_2_O, and FeOOH at a current density of 2000 mA g^−1^.


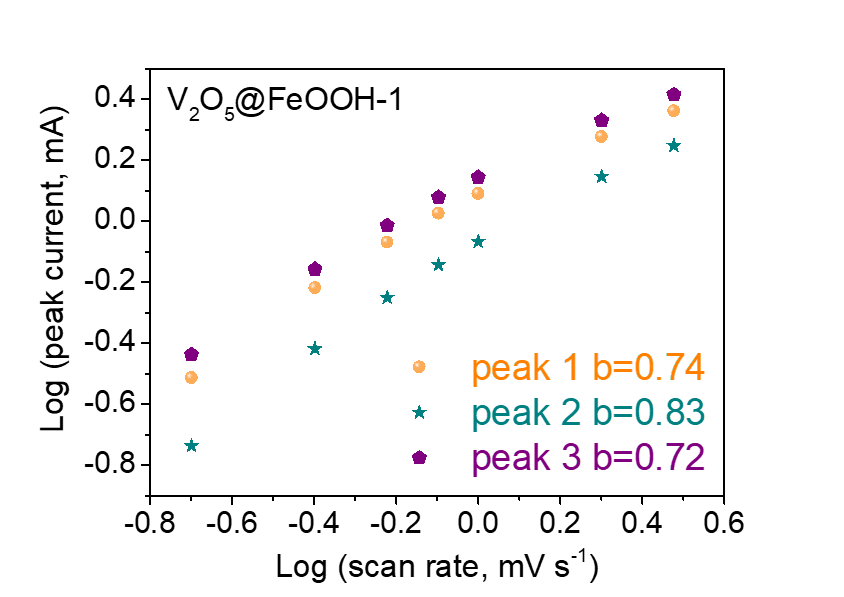


**Figure S14.** b value determined by the relationship between scan rate and peak current.

**The calculation details for electrochemical kinetics and quantitative analyses:**

In scan-rate-dependent CV curves, the current ($i$) at a specific potential ($\text{V}$) can be described as a summation of capacitive-controlled effect ($k_{1}v$) and diffusion-controlled reaction ($k_{2}v^{1/2}$) based on the equation:

$i(V)=k_{1}v+k_{2}v^{1/2}$ (1)

By plotting $i(V)/v^{1/2}$ vs. $v^{1/2}$ at different potentials, the values of $k_{1}$ (slope) and $k_{2}$ (intercept) from the straight lines can be calculated. After integration of the enclosed CV area, the amount of stored charge from different energy storage modes can be distinguished.


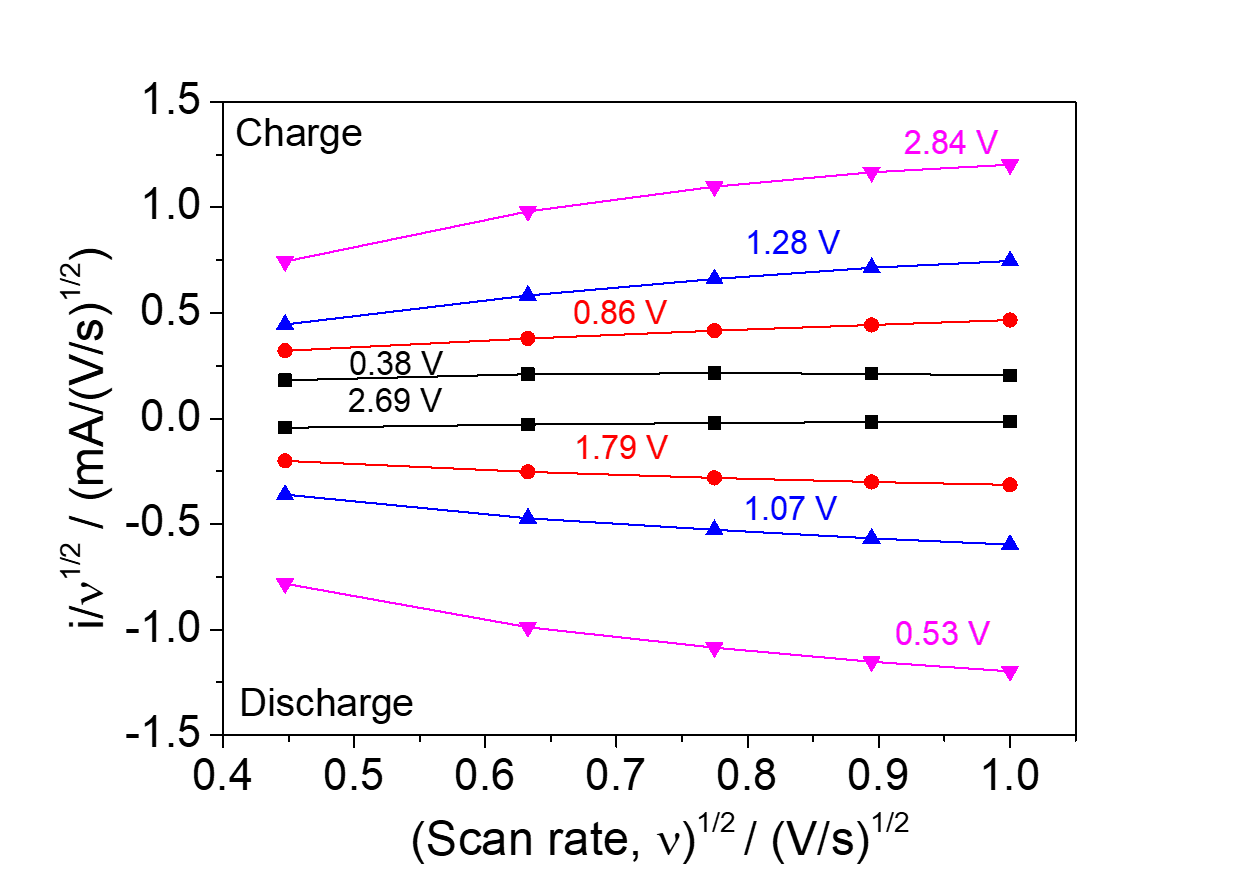


**Figure S15.** Plots of $v^{1/2}$ vs. $i/v^{1/2}$ used for calculating constants k_1_ and k_2_ at different potentials.

**
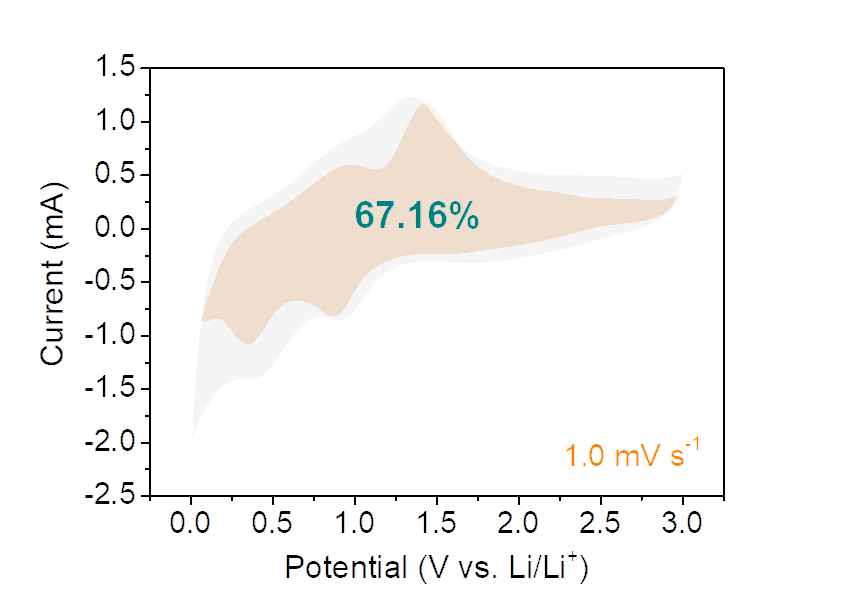
**

**Figure S1****6.** Capacitive contribution to charge storage of V_2_O_5_@FeOOH-1 at 1.0 mV s^−1^.


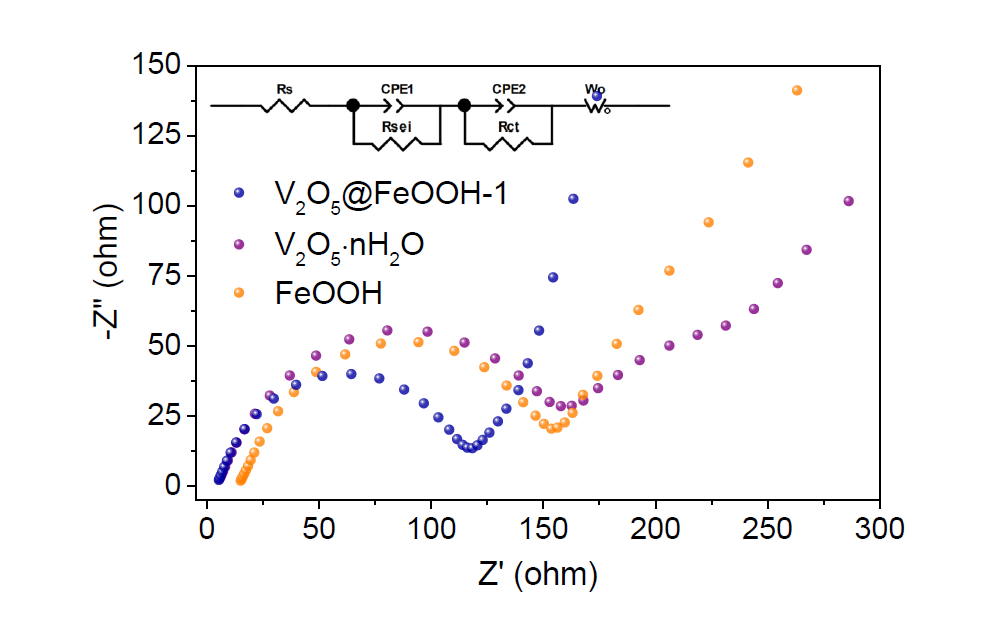


**Figure S17.** Nyquist plots of V_2_O_5_@FeOOH-1, FeOOH, and V_2_O_5_·nH_2_O, inset: the equivalent circuit for electrochemical impedance spectra of electrode materials.

**The calculation details for lithium diffusion coefficient:**

The lithium diffusion coefficients are calculated according to the following equation:

*$D_{Li^{+}}=\frac{R^{2}T^{2}}{2A^{2}n^{4}F^{4}C^{2}\sigma^{2}}$*

where$D_{Li^{+}}$ is the diffusion coefficient, $R$ is the gas constant, $T$ is the absolute temperature, $A$ is the surface area of the anode, $n$ is the total number of electrons transferred, $F$ refers to the Faraday constant, $C$ is the concentration of Li ions in the solid and $\sigma$ is the Warburg factor. The relationship between $\sigma$ and $Z'$ (real component of the impedance) in the low-frequency region is expressed as the following equation:

*$Z'=R_{D}+R_{L}+\sigma\omega^{-1/2}$*

Where *$\omega=2\pi f$* ($f$ is the frequency in low frequency region).


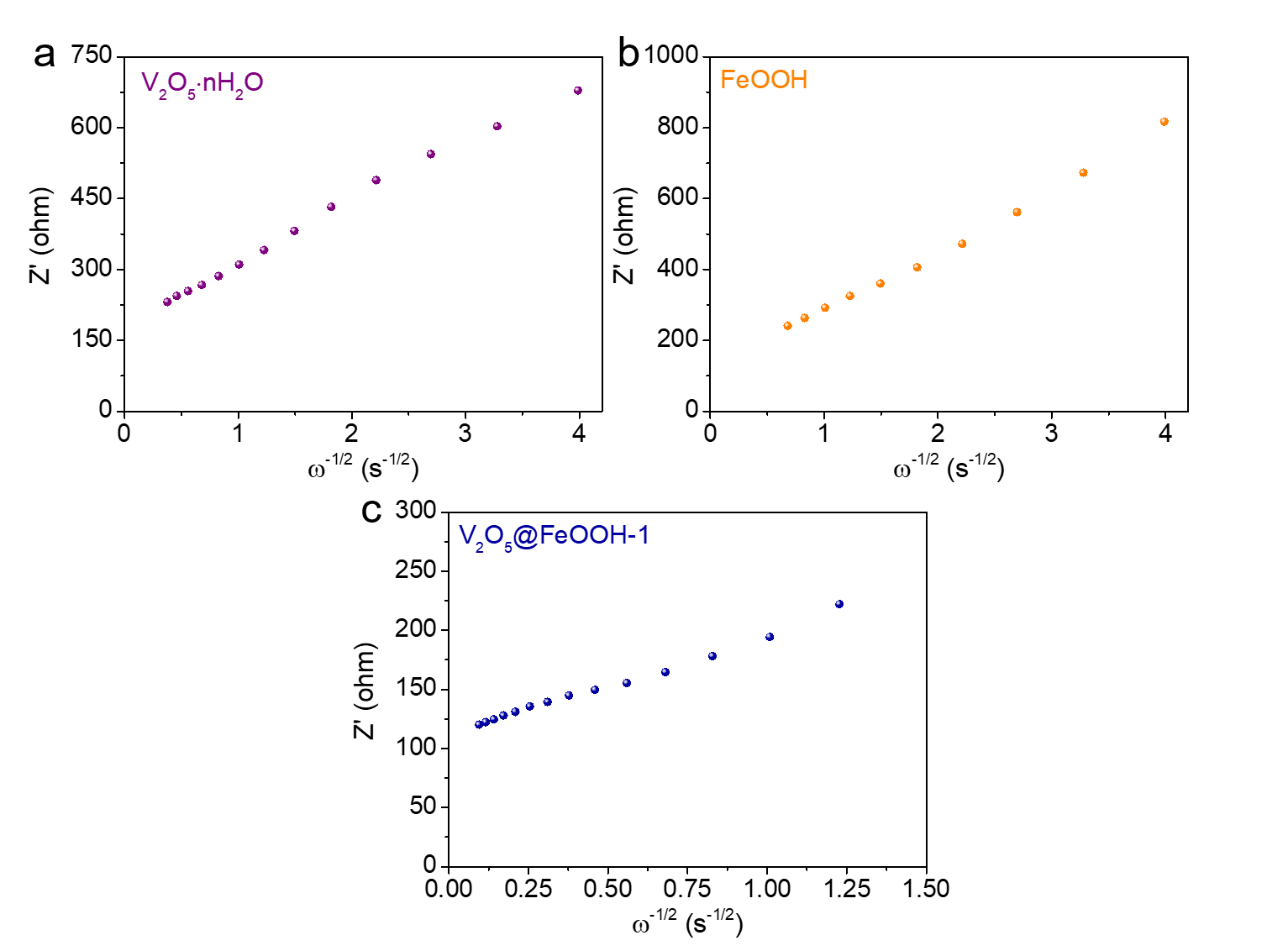


**Figure S18.** The relationships between $\omega^{-1/2}$ and $Z'$ in the low-frequency region of (a) V_2_O_5_·nH_2_O, (b) FeOOH, and (c) V_2_O_5_@FeOOH-1.

**Table S1.** The molar ratio of V and Fe for [V_2_O_5_@FeOOH-1](mailto:V2O5@FeOOH-1), [V_2_O_5_@FeOOH-2](mailto:V2O5@FeOOH-1), and [V_2_O_5_@FeOOH-5](mailto:V2O5@FeOOH-0.2) calculated based on XPS results.

|  | [V_2_O_5_@FeOOH-1](mailto:V2O5@FeOOH-1) | [V_2_O_5_@FeOOH-2](mailto:V2O5@FeOOH-1) | [V_2_O_5_@FeOOH-5](mailto:V2O5@FeOOH-0.2) |
| --- | --- | --- | --- |
| Molar ratio of V:Fe | 5.70:1 | 2.58:1 | 1.58:1 |

**Table S2.** Fitted equivalent resistances for EIS results of V_2_O_5_@FeOOH-1, FeOOH, and V_2_O_5_·nH_2_O.

|  | R_s_ (Ω) | R_SEI_ (Ω) | R_ct_ (Ω) |
| --- | --- | --- | --- |
| V_2_O_5_@FeOOH-1 | 6.1 | 12.3 | 114.7 |
| FeOOH | 12.4 | 114.2 | 125.5 |
| V_2_O_5_·nH_2_O | 3.2 | 133.4 | 181.0 |

| Materials | Morphology | Cycling performance | | |  | Rate capability | | Ref. |
| --- | --- | --- | --- | --- | --- | --- | --- | --- |
|  |  | Current density (mA g^−1^) | Cycling number | Capability (mAh g^−1^) | Cycling stability | Current density (mA g^−1^) | Capability  (mAh g^−1^) |  |
| V_2_O_5_@  FeOOH | Hollow nanoflower | 200 | 180 | 985 | 99% | 1000 | 714 | This work |
|  | Hollow nanoflower | 2000 | 300 | 494 | 99% | 3000 | 366 | This work |
| Si@V_2_O_5_ | nanosheet | 500 | 50 | 504 | / | 4000 | 510 | [1] |
| V_2_O_5_ | nanoparticle | 100 | 50 | 600 | 98% | 5000 | 410 | [2] |
| V_2_O_5_/SnO_2_ | Double-shelled nanocapsule | 250 | 50 | 673 | 95% | 2500 | 505 | [3] |
| V_2_O_3_ | nanobelt | 5000 | 1000 | 250 | 99% | 2000 | 600 | [4] |
| Fe_3_O_4_/VO*_x_*/graphene | nanowire | 100 | 30 | 519 | 99% | 2000 | 780 | [5] |
| V_2_O_5_-G | nanosheet | 1000 | 200 | 519 | 99.5% | 2000 | 375 | [6] |
| V_2_O_3_@carbon | nanobelt | / | / | / | / | 5000 | 205 | [7] |
| V_2_O_3_–OMC | nanoparticle | 100 | 180 | 536 | 98% | 2000 | 309 | [8] |
| V_2_O_3_/C NCs | nanoparticle | 200 | 180 | 780 | 99% | 2000 | 450 | [9] |
| LVO/C | Core-shell | 40 | 50 | 330 | 99% | 8000 | 250 | [10] |

**Table S3.** Comparison of lithium storage properties of various vanadium-based oxide materials.

**References**

[1] G. Carbonari, F. Maroni, A. Birrozzi et al., “Synthesis and characterization of Si nanoparticles wrapped by V_2_O_5_ nanosheets as a composite anode material for lithium-ion batteries,” *Electrochimica Acta*, vol. 281, no. pp. 676–683, 2018.

[2] O.B. Chae, J. Kim, I. Park et al., “Reversible Lithium Storage at Highly Populated Vacant Sites in an Amorphous Vanadium Pentoxide Electrode,” *Chemistry of Materials*, vol. 26, no. 20, pp. 5874–5881, 2014.

[3] J. Liu, H. Xia, D. Xue, L. Lu, “Double-Shelled Nanocapsules of V_2_O_5_-Based Composites as High-Performance Anode and Cathode Materials for Li Ion Batteries,” *Journal of the American Chemical Society*, vol. 131, no. 34, pp. 12086–12087, 2009.

[4] Y. Zhang, H. Wang, J. Yang et al., “Hydrogenated vanadium oxides as an advanced anode material in lithium ion batteries,” *Nano Research*, vol. 10, no. 12, pp. 4266–4273, 2017.

[5] Q. An, F. Lv, Q. Liu et al., “Amorphous Vanadium Oxide Matrixes Supporting Hierarchical Porous Fe_3_O_4_/Graphene Nanowires as a High-Rate Lithium Storage Anode,” *Nano Letters*, vol. 14, no. 11, pp. 6250–6256, 2014.

[6] X. Sun, C. Zhou, M. Xie et al., “Amorphous vanadium oxide coating on graphene by atomic layer deposition for stable high energy lithium ion anodes,” *Chemical communications*, vol. 50, no. 73, pp. 10703–10706, 2014.

[7] Y. Wang, H.J. Zhang, A.S. Admar et al., “Improved cyclability of lithium-ion battery anode using encapsulated V_2_O_3_ nanostructures in well-graphitized carbon fiber,” *RSC Advances*, vol. 2, no. 13, article 5748, 2012.

[8] L. Zeng, C. Zheng, J. Xi, H. Fei, M. Wei, “Composites of V_2_O_3_-ordered mesoporous carbon as anode materials for lithium-ion batteries,” *Carbon*, vol. 62, pp. 382–388, 2013.

[9] Y. Dong, R. Ma, M. Hu et al., “Polymer-pyrolysis assisted synthesis of vanadium trioxide and carbon nanocomposites as high performance anode materials for lithium-ion batteries,” *Journal of Power Sources*, vol. 261, pp. 184–187, 2014.

[10] C. Zhang, H. Song, C. Liu et al., “Fast and Reversible Li Ion Insertion in Carbon-Encapsulated Li_3_VO_4_ as Anode for Lithium-Ion Battery,” *Advanced Functional Materials*, vol. 25, no. 23, pp. 3497–3504, 2015.
